# Malignant rhabdoid tumor of kidney in an adult patient with positive family history of rhabdoid tumor: A case report and review of literature

**DOI:** 10.1016/j.ijscr.2023.109053

**Published:** 2023-11-13

**Authors:** Farhood Khaleghi mehr, Nasrollah Abian, Mandana Rahimi, Yasaman Moradi

**Affiliations:** a Department of Urology, Hasheminejad Kidney Center, School of Medicine, Iran University of Medical Sciences, Tehran, Iran; b Department of Urology, 5Azar Hospital, School of Medicine, Golestan University of Medical Sciences and Health Services, Gorgan, Iran; c Department of Pathology, Hasheminejad Kidney Center, School of Medicine, Iran University of Medical Sciences, Tehran, Iran

**Keywords:** Malignant rhabdoid tumor of kidney, Family history, Adult, Metastatic kidney cancer, Rhabdoid tumor, INI-1

## Abstract

**Introduction and importance:**

Malignant rhabdoid tumor of kidney (MRTK) is almost exclusive to children. Only 10 cases of adult MRTK have been reported. Here, we present a case of MRTK in an adult patient and discuss its clinical findings, diagnostic challenges, and treatment outcome. We also perform literature review on this issue.

**Case presentation:**

Our patient was a 29-year-old woman presented with fever and hematuria. She also mentioned atypical teratoid/rhabdoid tumor of cerebellum in her deceased child. Initial diagnostic work up led to left partial nephrectomy with the pathology report of high grade undifferentiated tumor. Early tumor recurrence necessitated left radical nephrectomy with extensive excision of adjacent tissues. Pathology for second specimen considering disease course and family history was MRTK. Even though chemotherapy was administered, she died few months later due to multiple metastases.

**Clinical discussion:**

Although diagnosis is challenging in all 11 reported cases –including our case- of adult MRTK, immunohistochemistry (i.e., negative reaction for INI-1) in conjunction with clinical and radiological findings are the main tool to reach diagnosis. Treatment options are much more diverse, ranging from surgery to immunotherapy, tyrosine kinase inhibitors, chemotherapy, and combination of these modalities. Prognosis remains dismal with the mean survival period of 7 months.

**Conclusion:**

Although extremely rare, MRTK might happen in adults. We report the first case of adult MRTK with positive family history of rhabdoid tumor of CNS, underscoring the importance of family history in reaching the diagnosis and highlighting the role of genetics in this rare disease.

## Highlights

• Malignant rhabdoid tumor of kidney may happen in adult patients.
• IHC must be interpreted along with clinical findings and imaging to reach diagnosis.
• Higher degree of suspicion is required to diagnose and treat these tumors as soon as possible.
• Family history might be very helpful in reaching the diagnosis, as in our case.
• Prognosis remains dismal despite of aggressive treatments.

## 1 Introduction

Malignant rhabdoid tumor of kidney (MRTK) is an uncommon and aggressive malignancy, primarily affecting infants and young children [1]. Its occurrence in adults is exceedingly rare, making diagnosis and management challenging. Here, we present a case of MRTK in an adult woman with a family history of atypical teratoid/rhabdoid tumor of cerebellum in her deceased child and discuss clinical presentation, diagnostic challenges, and treatment options. We also provide a literature review on MRTK in adults. Our case report has been reported in line with the SCARE criteria [2].

## 2 Presentation case

**Fig. 1:**
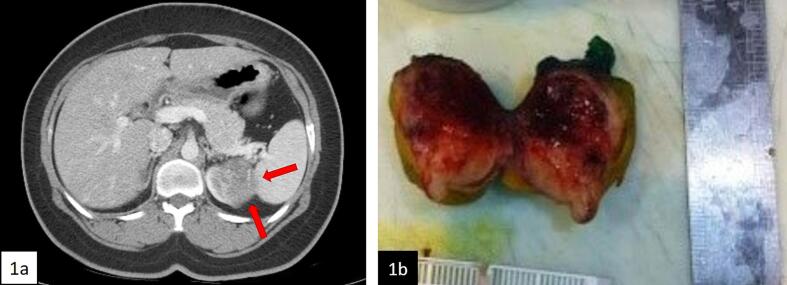
a. Abdominopelvic CT scan showing 5 cm mass in upper pole of left kidney with moderately ill-defined border (red arrows). b. Partial nephrectomy specimen: a creamy rubbery mass with foci of hemorrhage and necrosis. (For interpretation of the references to colour in this figure legend, the reader is referred to the web version of this article.)

A 29-year-old woman presented with mild fever and gross hematuria. She had no urinary symptoms, nor any significant past medical or surgical history. She mentioned her deceased child at 2 years of age due to atypical teratoid/rhabdoid tumor of cerebellum. Physical exam was normal except for fever (*T* = 38.2 °C). Sepsis work up yielded no infectious source. Approaching to hematuria, computed tomography (CT) revealed a 5 cm enhancing mass in left kidney (Fig. 1a) but no distant metastasis was observed. Urine cytology and cystoscopy was normal. Considering possibility of renal cell carcinoma (RCC), she underwent left partial nephrectomy (Fig. 1b).

**Fig. 2:**
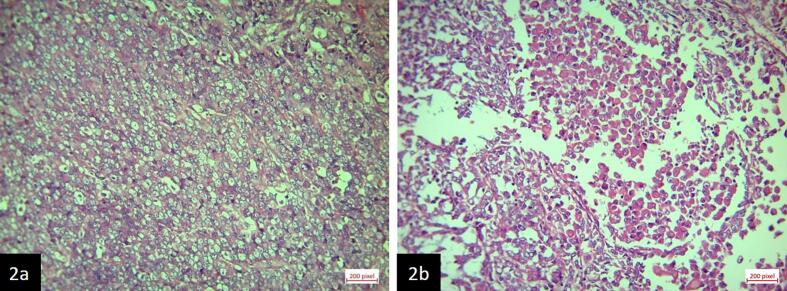
Microscopic examination. a. neoplastic tissue composed of diffuse proliferation of atypical cells with moderate to severe pleomorphism, vesicular nuclei, prominent nucleoli, and frequent mitotic figures (×40). b. Rhabdoid differentiation in tumor cells.

**Fig. 3:**
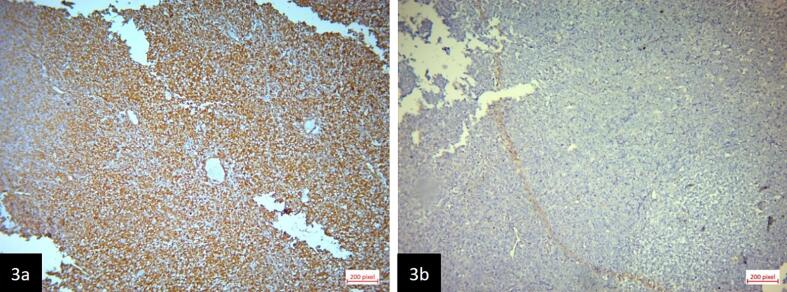
Immunohistochemical study. a. Strong and diffuse positive reaction for vimentin (×20). b. Negative reaction for INI-1 (×20).

Microscopic examination showed diffuse proliferation of high grade atypical cells with frequent mitotic figures and areas of rhabdoid differentiation (Fig. 2). Immunohistochemistry examinations were diffusely positive for vimentin; focal positive for SMA, CD56, and NSE; and negative for Pan CK, CEA (polyclonal), GATA-3, P63, AMACR, PAX-8, Melan-A, Myogenin, Myo-D1, OCT3/4, CD45, CD99, Cam5.2, INI-1, Desmin, S100, WT1, and Cam5.2 (Fig. 3). Pathology report was compatible with high grade undifferentiated tumor with rhabdoid feature. Surgical margins were free from tumor.

**Fig. 4:**
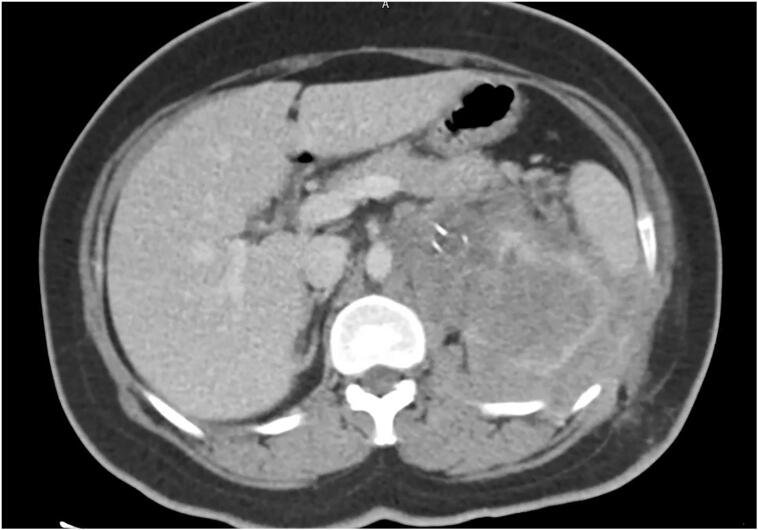
CT scan showing tumor recurrence as an infiltrative mass in the left kidney extending to renal hilum, peritoneum, psoas muscle, and abdominal wall.

She was referred to oncologist for adjuvant chemotherapy but before starting chemotherapy, she came to emergency department with fever and severe left flank pain. She was admitted with the impression of pyelonephritis but CT scan showed an infiltrative mass in the upper pole and interpolar part of left kidney extending to renal hilum, in favor of tumor recurrence (Fig. 4), and small single metastasis in the lung. A multidisciplinary meeting (MDM) was held with the presence of urologists, radiologists, pathologists, and oncologists to choose the best treatment plan. Considering her positive family history and reviewing her clinical and imaging findings, palliative left radical nephrectomy through retroperitoneal approach, i.e., flank incision (same as previous surgery), with chemotherapy afterwards was supported by all specialists during MDM.

Therefore, left radical nephrectomy was performed concomitant with adrenalectomy, lymphadenectomy, splenectomy, and excising parts of lower ribs, diaphragm, psoas muscle, and abdominal wall. Considering history of atypical teratoid/rhabdoid tumor of cerebellum in her daughter, aggressive behavior of tumor, and negative INI-1 on IHC, pathology report for the second surgery was MRTK. Ipsilateral adrenal gland and lymph nodes were involved and extension of tumor to peritoneum, ribs, and muscles was seen. Spleen remained from tumor.

Chemotherapy with 3 cycles of ifosfamide and doxorubicin was administered. At 3-months follow-up CT scan, the patient had no sign of disease progression. After taking the fourth cycle, and before starting the fifth one, the disease-progression showed itself by pathologic pelvic bone fracture; leading to diagnosis of multiple bone and visceral metastasis which succumbed her to death 6 months after her first surgery.

## 3 Discussion

While MRTKs comprise 1.8 % of renal malignancies in children [1], only 11 cases of MRTK in adults (including our case) have been reported in English literature. Table 1 summarizes important data from these publications. Although no sex or age predilection can be inferred from these cases, MRTK's tendency to occur in left side in noteworthy (9 out of 12 kidneys –including our case). Half of the patients had metastasis at the time of diagnosis with lungs being the most common site. Overall survival has been varied from few days up to more than 18 months.

**Table 1:** Reports of malignant rhabdoid tumor of kidney in adults in English literature.

Author/year	Sex/Age (years)/Side	Max Tumor diameter (cm)	Lymph node involvement	Distant metastasis at diagnosis	Main method for diagnosis	Treatment modalities	Outcome after therapy	Overall Survival
Lowe, et al./1990	Female/32/Right	5 cm	–	No	IHC	RN	Multiple metastases within 3 months	Not mentioned
Ebbinghaus, et al./1995	Male/56/Left	8 cm	–	Yes (lung)	IHC	Chemotherapy + IFN-α + IL-2 (biopsy proven diagnosis)	Regression of all pulmonary nodules except one, Shrinkage of primary tumor into half its size	+ 18 months survival
Peng, et al./2003	Female/38/Left	8 cm	Yes	No	IHC	RN + IL-2 + Chemotherapy	Multiple metastases within 2 weeks	5 months
Zhao, et al./2013	Male/5/Left	5 cm	Yes	Yes (lung)	IHC	RN	Not specifically mentioned (patient had metastasis before surgery)	+ 10 months
Podduturi, et al./2014	Male/60/Left	3.5 cm	Yes	Yes (lung)	IHC	RN	Deceased due to surgical complications	Few days
Okumura, et al./2019	Female/79/Left	6 cm	Yes	Yes (bone)	IHC	TKI (biopsy proven diagnosis)	Disease progression (primary tumor and metastases)	5 months
Ayari, et al./2019	Male/65/Right	5.5 cm	Yes	No	IHC	No treatment (biopsy proven diagnosis)	Deceased before starting any treatment	Few days
Han, et al./2021	Male/57/Left	7 cm, 4.5 cm	Yes	No	IHC, Next-generation sequencing	RN	Recurrence after 1 year	12 months
Goel, et al./2021	Female/65/Left	4.8 cm	No	No	IHC	RN (with splenectomy and distal pancreatectomy) + TKIs	Recurrence at tumor bed after 6 months	8 months
Walls, et al./2022	Male/21/Bilateral	5.4 cm, 2.5 cm	Yes	Yes	IHC	Chemotherapy (biopsy proven diagnosis)	Disease progression	3 months

IHC: immunohistochemical study, RN: radical nephrectomy, IFN: interferon, IL: interleukin, TKI: tyrosine kinase inhibitor.

The clinical presentation of MRTK in adults is similar to RCCs. According to previous cases, most common presenting symptoms were flank pain, gross hematuria, weight loss, and symptoms related to metastasis; all of which can be implicated to RCCs either. Our patient was presented with fever and gross hematuria; similar to aforementioned symptoms, fever can be related to paraneoplastic feature of RCC too. On the other hand, radiological findings can barely distinguish MRTK from RCC. Although irregular borders of the tumor and lymph node metastasis have been proposed as indicators of MRTK in children [3], there is still dearth of evidence to support this claim in adult MRTK. Reviewing our patient's first CT scan retrospectively, border of the tumor was moderately ill-defined (Fig. 1a, red arrows) but no other specific radiological finding, such as lymphadenopathy, could be found. Simply put, there are no clinical and radiological findings to differentiate MRTK from other renal tumors.

It seems that pathological assessment is our main armamentarium to diagnose MRTK, but microscopic evaluation alone can be misleading. MRTK was first considered to be a rhabdomyosarcomatoid variant of Wilms tumor but as no myogenic differentiation has been found in MRTK, it was later considered a distinct type of malignant renal mass [4]. Today, immunohistochemistry (IHC) and genetic/molecular analysis play a key role in MRTK diagnosis as this tumor is characterized by the loss of INI1 protein expression due to alterations in the SMARCB1 gene [1,5]. All of the reported cases of adult MRTK used IHC to reach diagnosis and one case applied genetic/molecular analysis to confirm it as well (Table 1).

The misperception of primary pathologic result has happened to our case as it was happened in the case of Han et al. [5]. However, reviewing clinical outcome necessitated revision in pathological findings in both cases. Paying attention to tumor's clinical behavior and patient's positive family history (i.e. atypical teratoid/rhabdoid tumor of cerebellum in patient's deceased child) in addition to IHC results, helped our pathologist to reach the diagnosis. Thus, while pathological assessments are our main tool to reach the diagnosis of adult MRTK, they are needed to be interpreted in the context of clinical findings.

Unlike previous articles where MRTK showed itself as a sporadic tumor, our case is the only case of adult MRTK with a family history of malignant rhabdoid tumor in a first degree relative; this is one of the highlights of our case report underscoring the role of genetics in MRTK.

No specific conclusion can be inferred from current data regarding best treatment of adult MRTK. Reported treatment modalities included surgery, immunotherapy (IL-2), tyrosine kinase inhibitors, chemotherapy, and combination of these modalities (Table 1). While chemotherapy is one of the major steps of therapy in pediatric MRTK [6], it is not widely administered in adult one; only 3 cases of adult MRTK were treated with chemotherapy in previous studies. More than half of the reported cases were subjected to radical nephrectomy and disease progression happened in almost all of those cases between 2 weeks to 12 months after surgery. Our case is the first case where partial nephrectomy was performed as initial step. Despite free surgical margins, we encountered tumor recurrence in kidney and solitary lung metastasis one month after surgery. Considering patient's severe pain, radical nephrectomy and splenectomy with extensive margin resection was performed to relieve symptoms and to decrease disease burden before starting chemotherapy.

Despite different treatment modalities, the prognosis of adult MRTK remains dismal, with the mean survival period of 7 months. Regardless of initial promising results at 3-months follow up imaging, multiple metastasis occurred in our patient 6 months after initial presentation which led to her death.

## 4 Conclusion

MRTK is an extremely rare malignancy, especially in adults. Being mindful regarding every small details in clinical and pathological findings seems to be the only promising way to reach the diagnosis. We reported the first case of adult MRTK with positive family history of rhabdoid tumor in the patient's first relative. Further research is warranted to establish standardized treatment guidelines and hopefully improve outcomes for this aggressive tumor.

## Abbreviations

MRTK: malignant rhabdoid tumor of kidney
CT: computed tomography
RCC: renal cell carcinoma
IHC: immunohistochemistry
MDM: multidisciplinary meeting

## Consent

Written informed consent was obtained from the patient for publication of this case report and accompanying images. A copy of the written consent is available for review by the Editor-in-Chief of this journal on request.

## Ethical approval

This case report is exempt from ethical approval due to the nature of the article (a retrospective description of a rare disease), as per the ethical review board at our institution.

## Funding

This research did not receive any specific grant from funding agencies in the public, commercial, or not-for-profit sectors.

## Author contribution

Study concept: Farhood Khaleghimehr

Data collection: Farhood Khaleghimehr, Nasrollah Abian,

Data interpretation: Nasrollah Abian, Mandana Rahimi, Yasaman Moradi

Writing the paper: Nasrollah Abian

Revision: Farhood Khaleghimehr

## Guarantor

Farhood Khaleghi mehr, Nasrollah Abian.

## Research registration number

Although pretty rare, our case report is not “First in Man”.

## Conflict of interest statement

No conflicts of interest.
